# Molecules affecting hypothalamic control of core body temperature in response to calorie intake

**DOI:** 10.3389/fgene.2012.00184

**Published:** 2012-10-05

**Authors:** Tamas Bartfai, Bruno Conti

**Affiliations:** ^1^Department of Chemical Physiology, The Scripps Research InstituteLa Jolla, CA, USA; ^2^Department of Molecular and Integrative Neurosciences, The Scripps Research InstituteLa Jolla, CA, USA

**Keywords:** core body temperature, calorie restriction, hypothalamus, neuropeptides, GPCR, homeostasis, warm-sensitive neurons

## Abstract

Core body temperature (CBT) and calorie intake are main components of energy homeostasis and two important regulators of health, longevity, and aging. In homeotherms, CBT can be influenced by calorie intake as food deprivation or calorie restriction (CR) lowers CBT whereas feeding has hyperthermic effects. The finding that in mice CBT prolonged lifespan independently of CR, suggested that the mechanisms modulating CBT may represent important regulators of aging. Here we summarize the current knowledge on the signaling molecules and their receptors that participate in the regulation of CBT responses to calorie intake. These include hypothalamic neuropeptides regulating feeding but also energy expenditure via modulation of thermogenesis. We also report studies indicating that nutrient signals can contribute to regulation of CBT by direct action on hypothalamic preoptic warm-sensitive neurons that in turn regulate adaptive thermogenesis and hence CBT. Finally, we show the role played by two orphans G protein-coupled receptor: GPR50 and GPR83, that were recently demonstrated to regulate temperature-dependent energy expenditure.

## INTRODUCTION

Experimental work on calorie restriction (CR), core body temperature (CBT), and the insulin-like growth factor 1 (IGF1)/Insulin pathway point at energy homeostasis as an important regulator of health, longevity, and aging. The two main components of energy homeostasis are nutrient and temperature homeostasis. Each contributes to energy intake and energy expenditure, respectively, and in homeotherms, they are regulated primarily in the hypothalamus. Although nutrient and temperature homeostasis are typically investigated independently, there is a distinct relationship between them. Calorie intake can affect CBT, with feeding producing acute hyperthermic effects, whereas food deprivation as well as the controlled reduction of nutrient intake in CR, can induce longer lasting hypothermia (Rampone and Shirasu, 1964; Walford and Spindler, 1997; Smirnov and Kiyatkin, 2008). CBT response to calorie intake reduction is regarded as an adaptive mechanism, decreasing energy expenditure when nutrient availability is limited. This mechanism may have evolved to prolong survival until food became available, and at least under controlled experimental conditions of CR, it contributes to increased lifespan. With some strain and diet specific differences, CR reduced CBT across species including mice, rats, primates, and humans (Duffy et al., 1989; Lane et al., 1996; Rikke et al., 2003; Soare et al., 2011). Work on transgenic mice with lowered CBT showed that even a modest (~0.5°C) but prolonged CBT reduction increased median lifespan of up to 20%. This was observed in animals on *ad libitum* diet and with a calorie intake similar to that of wild type (wt) littermates, demonstrating that the effects of CBT on longevity were independent from those of CR (Conti et al., 2006). These findings also suggested that the reduction of CBT occurring during CR may contribute to the effects of CR on longevity. Thus, the molecules and the pathways regulating CBT responses to calorie intake may be important regulators of aging.

Adaptive thermogenesis is controlled via the sympathetic nervous system (SNS), which influences heat production in the brown adipose tissue (BAT). BAT is a specialized tissue responsible for producing heat for adaptive thermogenesis by dissipating the mitochondrial proton gradient via the uncoupling protein 1 (UCP1). In rodents and human infants, BAT has been shown to be the major source of induced heat (Cannon and Nedergaard, 2004). In addition to BAT, the SNS can also influence CBT by affecting heat production in the skeletal muscles and the liver, as well as by restricting heat dissipation via regulation of peripheral vasoconstriction.

**FIGURE 1:**
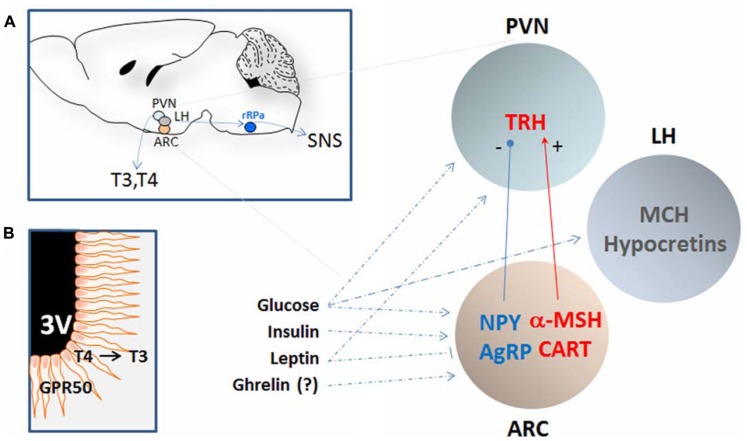
**Schematic representation of the hypothalamic nuclei and the neuropeptides that regulates calorie intake and energy expenditure by affecting core body temperature.** **(A)** The approximate location of the periventricular nucleus (PVN), the arcuate nucleus (ARC), and the lateral hypothalamus (LH) are shown in a sagittal section of the mouse brain (left). These regions are in polysynaptic contact with the BAT (not shown) via the raphe pallidus (RPa) and can regulate CBT by affecting the sympathetic nervous system (SNS). In addition, CBT can also be affected by thyrotropin-releasing hormones (TRH) that determines the levels of circulating thyroid hormones T3 and T4. The PVN and the ARC contain neurons that can respond to nutritional state by modulating the level of specific orexigenic or anorexigenic neuropeptides. The orexigenic neuropeptide Y (NPY) and agouti-related protein (AgRP) are elevated during calorie restriction or food deprivation and are strong stimulator of appetite and reduce CBT by acting on the SNS as well as by reducing the level of TRH in the PVN. In contrast, the anorexigenic peptide α-melanocyte-stimulating hormone (α-MSH), and possibly the cocaine-and amphetamine-regulated transcript (CART), stimulates thermogenesis. The LH is also an area important in the regulation of energy homeostasis and the melanin-concentrating hormone (MCH) and the hypocretins (Hcrt) can contribute to CBT elevation at least indirectly by increasing the locomotor activity associated with food seeking behavior and possibly via BAT activation. Neurons in these nuclei are sensitive to changes in the level of glucose, leptin, insulin, and possibly ghrelin all positively correlated to CBT. **(B)** Schematic representation of tanycytes cells lining the third ventricle (3V) and projecting into the hypothalamus. These cells can contribute to the regulation of CBT in response to nutrient signals in at least two ways: (1) by producing of T3 from T4 thus increasing its local concentration and in doing so inhibiting the synthesis of TRH even when circulating T3 levels are low; (2) by expressing GPR50, an orphan G-coupled protein receptor proposed to serve as a regulator of adaptive thermogenesis in response to nutrient signals. See text for details and references.

**FIGURE 2:**
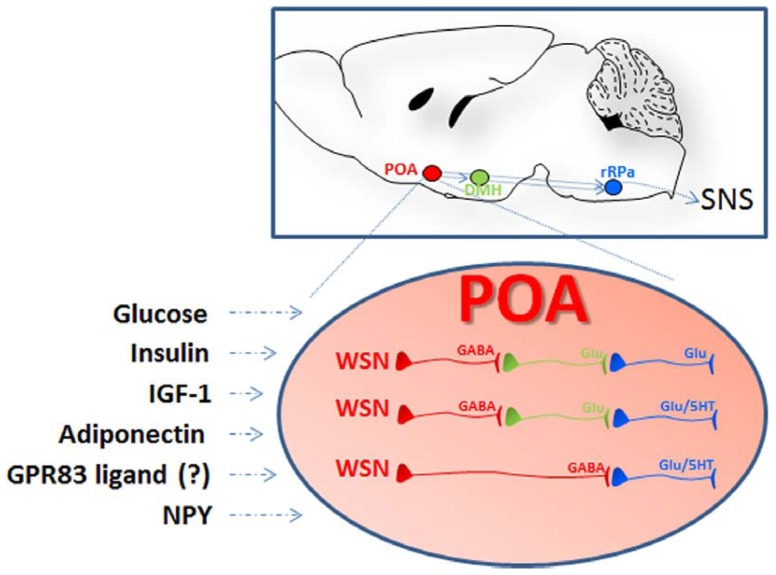
**Schematic representation of the localization and the organization of the nuclei and the cells participating to central thermoregulation.** Lesions studies indicate that the hypothalamic preoptic area (POA) exerts the function of a bona fide thermostat allowing sensing and proper thermoregulatory responses to local as well as peripheral temperature changes (afferent pathways are not shown). In addition to respond to changes in temperature the POA can also sense nutrient signals: POA injection of insulin, IGF-1, and adiponectin were followed by hyperthermia via BAT activation while treatment with NPY or downregulation of the G-protein coupled receptor 83 (GPR83) induced hypothermia. The receptors for some of these ligands, as well as GPR83, were demonstrated in the POA warm-sensitive neurons (WSN). These specialized GABAergic cells exert a tonic inhibition on the raphe pallidus (aRP) either directly or through neurons in the dorsomedial hypothalamus (DMH) and control thermogenesis by activation of brown adipose tissue (BAT), muscular shivering, or the regulation of vasodilation (scheme adapted from Morrison and Nakamura, 2011). WSN are one component of the POA thermoregulatory neurocircuitry that comprises also temperature insensitive and cold-sensitive neurons that might also participate in the POA responses to nutrients (not shown in this scheme).

This review will focus on signaling molecules demonstrated in mouse or in rat to be produced by and/or to act on two hypothalamic regions pivotal in the regulation of temperature or nutrient homeostasis, and that are in polysynaptic contact with the BAT (Elmquist et al., 2005). One such region includes the paraventricular (PVN), the arcuate (ARC), and the lateral hypothalamic (LH) nuclei (**Figure 1**); another is the preoptic area (POA; **Figure 2**).

The PVN, ARC, and LH express neuropeptides and their receptors, which together regulate feeding in addition to influencing CBT. These peptides include the neuropeptide Y (NPY), the cocaine- and amphetamine-regulated transcript (CART), the agouti-related protein (AgRP), the α-melanocyte-stimulating hormone (α-MSH), the melanin-concentrating hormone (MCH) and the thyrotropin-releasing hormone (TRH). These hypothalamic regions and neuropeptides are only reviewed here for their role in temperature homeostasis, and we refer to comprehensive reviews for their role in feeding (Elmquist et al., 2005; Morton et al., 2006; Gao and Horvath, 2007; Sanchez-Lasheras et al., 2010).

The POA contains temperature sensitive neurons that are pivotal in the sensing and the regulation of CBT (Hammel et al., 1960; Nakayama et al., 1963; Boulant, 2000). Among them are the warm-sensitive neurons (WSN), which are GABAergic neurons that exert a tonic inhibition on the dorsomedial hypothalamus (DMH) and the raphe pallidus (rRPa), both of which can activate spinal sympathetic and somatic motor circuits to drive adaptive thermogenesis in BAT (Morrison and Nakamura, 2011). WSN are typically investigated for their role in regulating fever or response to peripheral (skin) and local changes in temperature. However, electrophysiological studies and more recent molecular characterization have demonstrated that these specialized cells also respond to nutrient signals including glucose, insulin, and adiponectin (Silva and Boulant, 1984; Sanchez-Alavez et al., 2010; Eberwine and Bartfai, 2011; Klein et al., 2011). Evidence that these and other peripheral nutrient signals may contribute to CBT regulation via their action in the PVN, the ARC and the LH will also be summarized. Finally, we will discuss the role of the two orphan G protein-coupled receptors (GPCRs) GPR50 and GPR83 that were recently proposed to mediate effects of yet unidentified endogenous signals on energy expenditure via CBT regulation.

## HYPOTHALAMIC OREXIGENIC AND ANOREXIGENIC PEPTIDES

### NEUROPEPTIDE Y

Neuropeptide Y is a 36 aa, C terminally amidated neuropeptide. NPY acts at five different GPCR type of NPY-receptors (Y1–Y5), and is found in the autonomic nervous system and the brain, where its expression is highest in the ARC (**Figure 1**). NPY is a strong stimulator of feeding: its expression and synaptic level in the ARC is associated with hunger and is elevated during food deprivation or CR. Importantly, central administration of NPY not only increased food intake but also caused hypothermia (-1 to -3°C), reducing metabolic rate (Stanley et al., 1986). Such effect is at least in part due to decreased SNS-mediated thermogenesis, resulting from the NPY-mediated presynaptic (auto) inhibition of noradrenaline release from neurons that contain both norepinephrine (NE) and NPY. This also leads to a lower level of thermogenesis in BAT. In addition, Y1 and Y5 postsynaptic receptors on brown adipocytes also counteract the effect of NE at beta 3 adrenoreceptors (Billington et al., 1991; Egawa et al., 1991; Walker and Romsos, 1993; Bouali et al., 1995; Currie and Coscina, 1995; Pedrazzini et al., 1998; Williams et al., 2001). The Y5 agonists increased feeding, reduced oxygen consumption and energy expenditure in rats, probably by acting on ARC and BAT. Furthermore, the Y5 subtype selective antagonist increased CBT and the transcription of UCP1 in the BAT of mice (Hwa et al., 1999; Mashiko et al., 2007). Working with cold-acclimated Siberian hamsters, (Pelz and Dark (2007) and Dark and Pelz (2008)) found that activation of Y1 induced a prolonged reduction in CBT similar to that observed during natural torpor. Finally, inhibition of Y1 (albeit not of Y5) in hamster or its downregulation (knock-down) with antisense oligodeoxynucleotides in rats produced a transient hyperthermia (Lopez-Valpuesta et al., 1996; Pelz and Dark, 2007; Dark and Pelz, 2008).

Since the hypothermic action of NPY was observed not only after its administration into the ARC or the PVN, but also the POA, it was proposed that NPY influenced the activity of thermoregulatory neurons (Currie and Coscina, 1995; Jolicoeur et al., 1995; Dark and Pelz, 2008). Molecular profiling showed that POA WSN express Y2 as well as the GPR83, an orphan receptor sharing homology to Y2 and found by one group to interact with NPY *in vitro* (Sah et al., 2007; Eberwine and Bartfai, 2011; Dubins et al., 2012). Interestingly, downregulation of GPR83 expression in the POA by shRNA was recently shown to reduce CBT (discussed below; Dubins et al., 2012).

### AGOUTI-RELATED PROTEIN AND α-MELANOCYTE-STIMULATING HORMONE

The neuropeptides AgRP and α-MSH are the endogenous antagonist and agonist, respectively, of the melanocortin receptors and the main ligands of the central melanocortin system (**Figure 1**). In the hypothalamus they are produced in the ARC, where AgRP co-localizes with NPY, and where the precursor of α-MSH, the pro-opiomelanocortin (POMC), is co-expressed with CART (discussed below). AgRP and α-MSH stimulate and inhibit appetite, respectively, to modulate nutrient intake, but they can also contribute to the regulation of energy expenditure and can influence CBT (reviewed in Cone, 1999; Robinson et al., 2000; Schwartz et al., 2000; Spiegelman and Flier, 2001; Fan et al., 2005). This action is mediated by the melanocortin 4 receptor (MC4R) subtype. Mice null for MC4R, or treated with MC4R antagonists, including AgRP, have reduced thermogenesis and fail to upregulate UCP1 in the intercapsular BAT when fed high fat diet or when exposed to cold (Ste Marie et al., 2000; Butler et al., 2001; Voss-Andreae et al., 2007). Conversely, administration of MC3/4R agonist into the ventricle or in the RPa increased temperature via BAT activation (Yasuda et al., 2004).

Double labeling with retrograde tracing experiments demonstrated a connection between BAT and MC4R neurons in the RPa, the PVN, and the DMH MC4R mRNA was also demonstrated in LH, VMH, as well as in the POA, suggesting that the α-MSH and AgRP regulate BAT thermogenesis by acting on several different neuronal circuits (Kishi et al., 2003; Liu et al., 2003).

The melanocortin system can also influence the circulating levels of thyroid hormones (TRH and T3/T4), which were increased or reduced by intracerebroventricular (i.c.v.) injection of α-MSH or AgRP, respectively (Fekete et al., 2000a, 2002a; Kim et al., 2000). This is achieved by direct innervation of both α-MSH and AgRP fibers in the ARC to the TRH neurons in the PVN (Legradi and Lechan, 1999; Fekete et al., 2004; see below for the thermoregulatory action of TRH and thyroid hormones).

Interestingly, the melanocortin system was also recently demonstrated to be modulated by the NAD^+^-dependent deacetylase Sirt1, the mammalian homolog of Sir2 which was implicated in mediating some of the lifespan prolonging effects of CR (Guarente and Picard, 2005; Coppari, 2012). Sirt1 is expressed in the hypothalamus and its pharmacological inhibition decreased AgRP neuronal activity, producing a reduced inhibition of POMC neurons that was MCR4-dependent. Similar results were observed in animals with ablation of Sirt1 in AgRP neurons: these mice displayed decreased sensitivity to ghrelin, food intake, and body weight (Dietrich et al., 2010).

### COCAINE- AND AMPHETAMINE-REGULATED TRANSCRIPT

The CART is a neuropeptide co-expressed with POMC in neurons in the ARC (**Figure 1**). CART primarily affects energy homeostasis through its anorexigenic action, and can possibly contribute to energy expenditure by influencing CBT. In the rat, i.c.v. injection of CART was followed by hypothermic effects that were reduced by exendin-9-39, an antagonist of the glucagon like peptide 1 receptor also demonstrated to mediate the hypophagic of CART (Skibicka et al., 2009). However, a different study showed that CART injected in the PVN induced the expression of UCP1 in the BAT, suggesting that CART may be capable of stimulating adaptive thermogenesis by mitochondrial uncoupling (Wang et al., 2000).

### MELANIN-CONCENTRATING HORMONE AND HYPOCRETINS

The LH is another region involved in the regulation of feeding and energy expenditure, and lesions to this nucleus caused hypophagia as well as hyperthermia (Teitelbaum and Epstein, 1962; Stevenson and Montemurro, 1963). Two distinct families of signaling molecules are recognized as main modulators of nutrient homeostasis and energy expenditure in the LH: the MCH and hypocretins (also known as Orexins; **Figure 1**). MCH is a powerful stimulator of feeding and its ablation results in a lean phenotype (Shimada et al., 1998). Hypocretins are neuropeptides involved in the regulation of sleep, wakefulness, and reward, and they are also able to increase appetite and CBT (de Lecea et al., 1998; Sakurai et al., 1998; Yoshimichi et al., 2001; Adamantidis and de Lecea, 2009; Bonnavion and de Lecea, 2010).

Retrograde labeling studies demonstrated a link between MCH neurons and hypocretin neurons and BAT innervation and activity (Oldfield et al., 2002; Cerri and Morrison, 2005). The level of UCP1 transcripts in brown adipocytes was increased in mice null for MCH, and this mutation normalized CBT in leptin-deficient animals (Segal-Lieberman et al., 2003). Consistently, UCP1 mRNA levels in BAT were reduced by MCH infusion (Ito et al., 2003). However, MCH and hypocretins have profound effects on locomotion, rendering the contribution of muscular activity to energy expenditure difficult to evaluate. In our experience, for instance, a deletion of up to 90% of hypocretin neurons reduced locomotion without affecting CBT (Conti et al., 2006).

### THYROTROPIN-RELEASING HORMONE

The TRH is recognized as an important regulator of energy metabolism (reviewed in Lechan and Fekete, 2006). TRH exerts this action mainly via the modulation of the hypothalamic–pituitary–thyroid (HPT) axis, regulating the level of the thyroid hormones thyroxine (T4) and triiodothyroxine (T3). TRH neurons in the PVN regulate the release of the pituitary thyroid-stimulating hormones (TSH) into the circulation that, in turn, act on the thyroid gland to release T3 and T4 (**Figure 1**). Importantly, thyroid hormones inhibit TRH secretion, providing a negative regulatory feedback onto the axis. Thyroid hormones have long been recognized as mediators of thyroid thermogenesis, a phenomenon mainly investigated as a peripheral event evoked via direct thyroid action on muscle cells, involving altered muscle cell Ca^2^^+^ homeostasis, and possibly UCP3 (reviewed in Silva, 2006). More recently, it was proposed that the mechanisms of T3-induced thermogenesis are central and involve the sympathetic activation of BAT, requiring the activation of the lipogenic pathway in the ventromedial hypothalamus (Cannon and Nedergaard, 2010; Lopez et al., 2010).

Thyrotropin-releasing hormone, as well as T3, are also important regulators of feeding, and experimental work indicated that both hormones can regulate thermogenesis in response to calorie intake. Fasting induced a fall in T3 and T4 levels and a reduction of TRH expression in the PVN, an effect at least in part due to decreased level of anorexigenic peptides (Spencer et al., 1983; Connors et al., 1985; Blake et al., 1991, 1992; Ahima et al., 1996; Legradi et al., 1998). These actions are mediated in two manners: by leptin acting directly on TRH neurons in the PVN, or by leptin acting indirectly by exerting opposite actions on α-MSH/CART and NPY/AgRP neurons localized in the ARC and projecting to the PVN (Ahima et al., 1996, 2000; Ahima, 2000; Nillni et al., 2000; Harris et al., 2001; Bjorbaek and Hollenberg, 2002; Perello et al., 2010). Fasting-induced reduction of TRH can be restored by i.c.v. injection of α-MSH or CART, which activate TRH neurons and stimulate hormone release (Fekete et al., 2000a,b; Kim et al., 2000; Nillni et al., 2000). Both AgRP and NPY can inhibit TRH neurons, reducing TRH transcript and circulating thyroid hormone levels. The NPY action is also mediated via Y1 and Y5 receptors (Fekete et al., 2001, 2002a,b; Costa-e-Sousa et al., 2011; Vella et al., 2011). Recently, it was reported that MC4R and NPY are both required for hepatic metabolism of T4 during fasting (Vella et al., 2011).

A distinct hypothalamic mechanism for the downregulation of the HPT axis is represented by local increase of T3 via fast-induced elevation of type 2 iodothyronine deiodinase in tanycytes, a group of ependymal cells that are located at the base of the third ventricle and extend into the hypothalamus (**Figure 1B**). During fasting, the type 2 deiodinase, D2 can convert T4 into the more potent T3 whose feedback inhibits the HPT axis, lowering the level of circulating thyroid hormones (Diano et al., 1998). Interestingly, these cells produce high level of GPR50, an orphan receptor also expressed in several hypothalamic nuclei and in pituitary neurons. GPR50 was recently shown to be a strong regulator of energy expenditure and thermogenesis in the context of the state of torpor (Bechtold et al., 2012; and see below).

Finally, TRH may also influence thermogenesis by direct action on POA temperature sensitive neurons and without affecting the HPT axis. Central injection of TRH was, in fact, capable of decreasing the activity of a fraction of WSN and increasing that of cold-sensitive neurons in the POA (Hori et al., 1988). This finding is consistent with the central hyperthermic effect of TRH, although its possible role in influencing CBT in response to nutrient intake remains to be investigated.

## PERIPHERAL NUTRIENT SIGNALS

### GLUCOSE

A central role of glucose in influencing CBT was first revealed by experiments in which i.c.v. injection of the glucose analog 2-DG was followed by reduced sympathetic activation of BAT and hypothermia (Freinkel et al., 1972; Egawa et al., 1989). Glucose-sensing neurons are found in most hypothalamic nuclei (Ritter and Dinh, 1994; Dunn-Meynell et al., 1998; Silver and Erecinska, 1998; Briski and Sylvester, 2001; Fioramonti et al., 2004; Wang et al., 2004; Yang et al., 2004) as well as at the brain stem level in the area postrema (AP), the nucleus of the solitary tract (NTS), the dorsal motor nucleus of the vagus (DMNX), and the basolateral medulla (BLM) (Adachi et al., 1984; Mizuno and Oomura, 1984; Ritter and Dinh, 1994; Yettefti et al., 1995; Dunn-Meynell et al., 1998; Dallaporta et al., 1999; Briski and Sylvester, 2001). It is possible to distinguish two categories of neurons depending on whether the elevation of extracellular glucose level has excitatory or inhibitory action on their activity (Anand et al., 1964; Adachi et al., 1984; Routh, 2002; Yang et al., 2004). Using mice with inactivation of the glucose transporter type 2 (Glut2), Mounien et al. (2010) demonstrated that the effects of glucose on thermogenesis are at least in part mediated via decreased leptin sensitivity of NPY and POMC expressing neurons in the ARC. These actions on the ARC may not be direct, but mediated by glucose-sensing neurons located in the LH, the dorsal vagal complex, and the basal medulla.

Interestingly, Glut2 neurons were also found in the DMH, an area that receives projections from POA WSN, suggesting the possibility that glucose may also influence adaptive thermogenesis via this neuronal circuitry. Finally, electrophysiological studies revealed that POA neurons, including a fraction of warm and cold-sensitive neurons, are sensitive to glucose (Silva and Boulant, 1984; **Figure 2**).

### LEPTIN

Leptin is a small protein produced by adipose tissue that acts peripherally as well as centrally to regulate appetite and energy expenditure (Campfield et al., 1995; Halaas et al., 1995; Pelleymounter et al., 1995). Mice null for leptin receptor or for the transcription factor STAT3, which is involved in leptin receptor signaling, are obese and have reduced CBT and oxygen consumption (Gao et al., 2004). Leptin-deficient mice spontaneously enter into torpor when deprived of food, a response that is prevented by leptin administration (Gavrilova et al., 1999). Conversely, in wt mice leptin reduced food intake, elevated CBT, and increased the sympathetic activation of BAT (Pelleymounter et al., 1995; Haynes et al., 1997).

The effects of leptin on energy expenditure and thermogenesis have mostly been investigated for leptin’s ability to regulate TRH either by direct action on PVN neurons, or by indirect action via inhibition of NPY/AgRP and stimulation of POMC/CART neurons in the ARC (Ahima et al., 1996, 2000; Ahima, 2000; Nillni et al., 2000; Schwartz et al., 2000; Harris et al., 2001; Bjorbaek and Hollenberg, 2002; Perello et al., 2010; see also Thyrotropin-Releasing Hormone).

A distinct mechanism of action for leptin-induced thermogenesis was also proposed to occur via stimulation of the release of the endogenous pyrogen interleukin-1β and prostaglandins (Luheshi et al., 1999) acting on POA and MPO neurons.

### GHRELIN

The gastrointestinal peptide ghrelin is a hunger-stimulating hormone produced mainly by specialized cells in the fundus of the stomach and the pancreas. Ghrelin promotes an increase in food intake and a reduction in energy expenditure, resulting in a positive energy balance and an increase in body weight (Tschop et al., 2000; Lawrence et al., 2002; Theander-Carrillo et al., 2006).

Definitive proof for a role of ghrelin in regulating CBT is still lacking since findings remain few and contrasting. Central i.c.v. injection of ghrelin was reported to not only to be able to provoke a transient reduction of CBT associated with decreased spontaneous activity, but also to promote a small but significant reduction of BAT temperature, which indicates that ghrelin may be capable of reducing energy expenditure by affecting temperature homeostasis (Lawrence et al., 2002; Yasuda et al., 2003; Tang-Christensen et al., 2004). A single case of severe hypothermia in humans subject to prolonged treatment with ghrelin was also reported (Wiedmer et al., 2011). When the same group further investigated the hypothermic effect of ghrelin in rodents, they found evidence that ghrelin could bind to axon terminals in the POA, but they did not see any effects on CBT when the peptide was injected i.c.v. or subcutaneously.

Findings that CBT reduction may not be one of the mechanisms by which ghrelin regulates energy expenditure also came from experiments using mice null for ghrelin *O*-acyltransferase (GOAT), the enzyme that catalyzes the octanoylation of ghrelin, that is a post-translational modification necessary for the biological activity of this peptide. CBT profiles in GOAT null mice were similar to that of their wt littermates in different nutritional states, including fasting, or when exposed to different ambient temperatures (Heppner et al., 2012).

Instead, two distinct studies suggest that ghrelin may have a role in fasting-induced torpor. One found that the torpor induced by food deprivation was more severe if animals were treated with ghrelin peripherally. These effects were lost in animals with chemical ablation of the ARC, or in mice null for NPY, but not in mice blocked in α-MSH pathway (Gluck et al., 2006). Another study found that mice null for pre-pro-ghrelin had increased sensitivity to fasting and lowered ambient temperature, resulting in a precipitous drop of CBT, impaired sleep pattern, and decreased survival (Szentirmai et al., 2009). However, such a phenotype was not observed in mice lacking ghrelin receptor, suggesting that additional ghrelin receptor subtypes may exist. In addition, some of the differences in these studies may be due to the distinct ambient temperature at which experiments were carried out, with the hypothermic effects of ghrelin reported only at 17–18°C, but not at 25°C, a value closer to thermo-neutrality.

### INSULIN/IGF-1

The pancreatic hormone insulin is the main regulator of peripheral glucose homeostasis and has been also investigated for its role as regulator of energy homeostasis in the central nervous system (Woods et al., 1979; Baskin et al., 1987; for recent reviews, see Plum et al., 2006; Belgardt and Bruning, 2010). Indeed, the insulin receptor (IR) is expressed in several brain regions, including the hypothalamus where it is abundant in the ARC (Havrankova et al., 1978; Werther et al., 1987; Marks et al., 1990). Pharmacological studies with central insulin injection, as well as elegant transgenic models of selective IR-ablation, showed that insulin can act centrally to cause reduced food intake, increased weight loss, and helped to regulate peripheral glucose homeostasis (Woods et al., 1979; McGowan et al., 1992a; Chavez et al., 1995; Bruning et al., 2000; Obici et al., 2002; Brown et al., 2006; Koch et al., 2008).

A role of insulin in regulating thermogenesis in response to feeding was proposed when it was observed that pharmacological inhibition of its secretion effectively attenuated diet-induced thermogenesis (Rothwell and Stock, 1981, 1986, 1988). Since either peripheral or central administration of insulin activated the SNS, the involvement of BAT in this response was promptly hypothesized (McCormack, 1982; Rothwell and Stock, 1986; Muntzel et al., 1995).

Injection of insulin into the hypothalamus had hyperthermic effects, increasing CBT and energy expenditure (Menendez and Atrens, 1991; McGowan et al., 1992a,b). This was proposed to occur via the insulin-mediated inhibition of the NPY/AgRP neurons expressing IR (Porte et al., 2002, 2005; Fekete et al., 2006; Mayer and Belsham, 2009).

The presence of IRs in the POA raised the possibility that insulin may influence thermogenesis by also acting on neurons in this region (Unger et al., 1989; Cardona-Gomez et al., 2000; Plum et al., 2005; van Baak, 2008). Central i.c.v. injection of insulin reduced the unit activity of POA neurons sensitive to peripheral changes in scrotum temperature, indicating that this hormone may modulate thermoregulatory responses by affecting these specialized cells (Wang and Lin, 1985). Recently, IR was demonstrated on at least a fraction of POA WSN, and electrophysiological studies on hypothalamic slices demonstrated that insulin acted directly on intrinsically WSN, inducing hyperpolarization and reducing their firing rate (Sanchez-Alavez et al., 2010). Retrograde transport and double labeling studies also demonstrated that the IR-positive WSN are GABAergic and project to the RPa (thus a likely synaptic connection to BAT was established). Finally, POA injection of insulin induced a specific, PI3K-involving and dose-dependent elevation of CBT mediated by stimulation of BAT (**Figure 2**).

A similar finding was reported for the IGF-1 (Sanchez-Alavez et al., 2011). Its receptor can be expressed on WSN and POA, and an injection of IGF-1 elicited a dose-dependent increase of CBT and activated BAT. Although the effects of IGF-1 on WSN activity remain to be demonstrated, the CBT effects of central IGF-1 were reduced in mice lacking neuronal IR. Since IGF-1 can also activate IR, the IR homodimers or the IGF-1R/IR heterodimers may contribute to the thermogenic action of IGF-1 (Sanchez-Alavez et al., 2011).

### ADIPONECTIN

Adiponectin is a protein hormone secreted by adipose tissue. It has insulin-sensitizing effects, and is an important regulator of metabolism in peripheral tissues, enhancing fatty acid oxidation and glucose uptake in muscle, and reducing hepatic glucose production (Berg et al., 2001, 2002; Fruebis et al., 2001; Yamauchi et al., 2001; Tomas et al., 2002; Shklyaev et al., 2003; Qi et al., 2004). The adiponectin receptors AdipoR1 and AdipoR2 are expressed in different brain regions such as the hypothalamus, where adiponectin is beginning to be investigated for its possible central effects (Yamauchi et al., 2003; Fry et al., 2006; Kos et al., 2007; Kubota et al., 2007; Coope et al., 2008; Guillod-Maximin et al., 2009; Psilopanagioti et al., 2009; Hoyda and Ferguson, 2010; Thundyil et al., 2011).

So far, only a limited number of studies have measured the effects of adiponectin on CBT and energy expenditure and these have reported contrasting findings. For instance, one group found i.c.v. injection of adiponectin recapitulated its peripheral effects and increased energy expenditure via BAT-induced thermogenesis (Qi et al., 2004). Another showed increased BAT UCP1 and rectal temperature following peripheral, but not central injection with adiponectin (Masaki et al., 2003). A third group instead reported that intravenous injection of adiponectin reduced BAT UCP1 mRNA and energy expenditure while exerting central orexigenic effects via direct action on the ARC (Kubota et al., 2007). The effects of central injections of adiponectin on calorie intake are also contrasting, reporting either pro-anorexigenic or pro-orexigenic effects, as well as no effects at all (Masaki et al., 2003; Kubota et al., 2007; Coope et al., 2008).

Since both AdipoR1 or AdipoR2 were recently found in a fraction of POA WSN we tested the effects of adiponectin on thermogenesis in mice null for either one of the adiponectin receptors (Eberwine and Bartfai, 2011; Klein et al., 2011). When injected locally into the POA of wt mice, adiponectin had thermogenic effects elevating CBT and fatty acid oxidation (measured as decreased respiratory exchange ratio). These effects were nearly abolished in mice lacking AdipoR1, and were only diminished in animals null for AdipoR2. It is possible that some of the contrasting findings may be due to differences in the experimental conditions used, or to the putative opposite roles that AdipoR1 and AdipoR2 were found to have on energy metabolism (Bjursell et al., 2007). Another confounding factor may be that the oligomer form of adiponectin used as the adiponectin monomer can oligomerize to form 3-mers that can further aggregate into 6-, 12-, or 18-mers (Pajvani et al., 2003). Kubota et al. (2007) reported that in mice, only 3- and 6-mers can enter the CSF from the circulation.

## TWO INTERESTING ORPHAN GPCRs WITH HYPOTHALAMIC EXPRESSION

The GPCRs are the favorite drug target class of the pharmaceutical industry and many of the most used and safest drugs are ligands to this class of receptors, including beta blockers, the antihistamines, and the D2 receptor antagonist antipsychotics to mention a few. The relative ease by which ligands to GPCRs are developed is the reason for excitement in the discovery of orphan GPCRs with physiologically and pharmacologically interesting and robust effects. Thus we examine into the effects mediated by GPR83 and GPR50, because it is likely that the validation of their role in integration of nutrient and energy homeostasis will lead to the development of useful drugs that affect feeding body weight and life span.

### GPR83

Profiling of WSN revealed that these cells express several orphan GPCRs (Eberwine and Bartfai, 2011). Among these is GPR83 (also known as GIR, GPR72, or JP05), originally identified as a stress–response element from a murine thymoma cDNA library treated with glucocorticoids and forskolin (Harrigan et al., 1989, 1991; Baughman et al., 1991), and subsequently shown to be highly expressed in several brain regions including the hypothalamus, the cortex, the thalamus, the hippocampus, and the amygdala (Pesini et al., 1998; Brezillon et al., 2001; Wang et al., 2001; Adams et al., 2003; Sah et al., 2005). GPR83 shares some homology with a variety of known peptide receptors, including the neuropeptide Y2 receptor. One study reported that NPY C-terminus fragments can bind and activate rat GPR83 with moderate affinity suggesting that GPR83 might participate in the regulation of nutrient intake (Sah et al., 2007).

Local downregulation of GPR83 in the hypothalamic POA, by injection of lentiviral vectors expressing a pool of shRNAs directed against all known isoforms of mouse GPR83 recently demonstrated its role in temperature homeostasis (Dubins et al., 2012). Reduction of POA GPR83 in the range of 30–50% caused a modest (0.15°C) but significant reduction of CBT, starting at day 4 post-treatment, that lasted at least until recording was stopped at day 18. CBT reduction was observed only in the dark period of the day, when the mice are active, and was not significant during the light-inactive phase. The downregulation of the expression GPR83 did not alter calorie intake, and animals treated with silencing GPR83 shRNA ate similarly to those treated with the non-silencing counterpart. However, the silencing shRNA treated group showed an increase in body weight gain that became significant 3 weeks after treatment and was associated with reduced hypothalamic receptor expression. This phenotype was similar to that observed in the long-lived transgenic mice, with reduced CBT achieved by producing heat through uncoupling neuronal mitochondria in the vicinity of WSN cells in the POA (Conti et al., 2006).

### GPR50

GPR50 is a GPCR recently demonstrated to play an important role in adaptive thermogenesis in response to calorie intake (Bechtold et al., 2012). It was originally cloned from human pituitary gland and termed melatonin-related receptor (MRR) for its homology with the melatonin receptors (Reppert et al., 1996; Dufourny et al., 2008, 2012). GPR50 does not bind to melatonin, and although it may dimerize with melatonin receptors (possibly influencing melatonin action) to date it remains an orphan receptor (Levoye et al., 2006). Expression of GPR50 is high in the hypothalamus, where it localized in the medial POA, the LH neurons of the dorsomedial nucleus, and in tanycytes (Reppert et al., 1996; Drew et al., 1998, 2001; Hamouda et al., 2007; Sidibe et al., 2010; Batailler et al., 2011; Bechtold et al., 2012).

When fed *ad libitum*, mice null for GPR50 (*Gpr50*^-/-^) showed a modest (~0.5°C) reduction of CBT, that like in the Hcrt-UCP2 mice and the GPR83 shRNA mice, respectively, was observed only during the dark-active part of the day. In response to 24 h food deprivation, CBT of *Gpr50*^-/-^ mice dropped up to 10°C. O_2_ consumption and CO_2_ production were also reduced, and mice entered a torpor-like state. The exact mechanisms by which GPR50 may affect thermogenesis remain to be elucidated. The experimental evidence collected thus far suggest that GPR50 can affect thermal responses to energy signals by directly reducing the responses to leptin and melanocortin during fasting in the ARC, and indirectly by suppressing TRH in the PVN, possibly normally inhibiting entry into a hypometabolic state (Bechtold et al., 2012). 

## SUMMARY

Lowered CBT increased lifespan and its value in homeotherms can be affected by calorie intake. Here we reviewed the current knowledge on the molecules and signals that mediate CBT responses to calorie intake as these may influence longevity and aging.

At least two hypothalamic regions are involved in mediating these responses. One is the region containing the ARC, the PVN, and the LH nuclei, which synthesize neuropeptides to regulate feeding, but are also able to affect CBT. The second is the POA, recognized for integrating and regulating peripheral as well as central temperature information, and containing temperature sensitive neurons that can also respond to nutrient signals. Both regions can activate the SNS and are in polysynaptic contact with BAT, a tissue specialized in heat production, and that in small animals, such as mice and rats, is the main contributor to thermogenesis. Within the ARC and the LH, the anabolic neuropeptides NPY, AgRP, and MCH are elevated during food deprivation, stimulating appetite and concomitantly reducing thermogenesis. In contrast, the catabolic neuropeptide α-MSH has opposite effects. In addition, AgRP and α-MSH were also proposed to regulate thermogenesis by inhibiting or stimulating the release of TRH from the PVN, thus influencing the level of circulating thyroid hormone.

Nutrient signals can influence thermoregulation by affecting the level of the aforementioned neuropeptides or by inhibiting the activity of WSN POA neurons. In particular, glucose, insulin, and leptin can act on neurons in the PVN and the ARC, stimulating the release of TRH and α-MSH, and reducing that of NPY and AgRP, promoting CBT increase. The POA was demonstrated to be responsive to glucose, insulin, IGF-1, NPY, and adiponectin, and some of these signals were demonstrated to modulate WSN activity. Two orphan GPCRs, GPR83 and GPR50, were also recently proposed to be instrumental in influencing CBT in response to calorie intake. GPR83 is elevated in the hypothalamus during CR, is expressed on WSN, and its local downregulation in the POA reduced CBT. GPR50, primarily expressed in tanycytes lining the third ventricle, appears to be an important regulator of the hypothermic response to fasting.

## Conflict of Interest Statement

The authors declare that the research was conducted in the absence of any commercial or financial relationships that could be construed as a potential conflict of interest.

## Abbreviations

α-MSH: α-melanocyte-stimulating hormone
AgRP: agouti-related protein
ARC: arcuate nucleus
BAT: brown adipose tissue
CART: cocaine- and amphetamine-regulated transcript
CBT: core body temperature
CR: calorie restriction
DMH: dorsomedial hypothalamus nucleus
DMNX: dorsal motor nucleus of the vagus
Glut2: glucose transporter type 2
GOAT: ghrelin *O*-acyltransferase
GPR83: G protein-coupled receptor
IGF-1: insulin-like growth factor 1
IR: insulin receptor
LH: lateral hypothalamus
MCH: melanin-concentrating hormone
MC4R: melanocortin 4 receptor
MRR: melatonin-related receptor
NE: norepinephrine
NPY: neuropeptide Y
NTS: nucleus of the solitary tract
POA: preoptic area
POMC: pro-opiomelanocortin
PVN: paraventricular nucleus
rRPa: raphe pallidus
SNS: sympathetic nervous system
TRH: thyrotropin-releasing hormone
TSH: thyroid-stimulating hormones
UCP1: uncoupling protein 1
WSN: warm-sensitive neurons
